# Diabetic striatopathy associated with nonketotic hyperglycemia

**DOI:** 10.1210/jcemcr/luag075

**Published:** 2026-04-29

**Authors:** Joseph Arguinchona, Tamra Ranasinghe, Jeffrey S Ross, Curtiss B Cook

**Affiliations:** Department of Endocrinology, Mayo Clinic Arizona, Scottsdale, AZ 85259, USA; Department of Neurology, Mayo Clinic Arizona, Scottsdale, AZ 85259, USA; Department of Radiology, Mayo Clinic Arizona, Scottsdale, AZ 85259, USA; Department of Endocrinology, Mayo Clinic Arizona, Scottsdale, AZ 85259, USA

**Keywords:** diabetic striatopathy, nonketotic hyperglycemic, hemichorea

## Image legend

**Figure 1:**
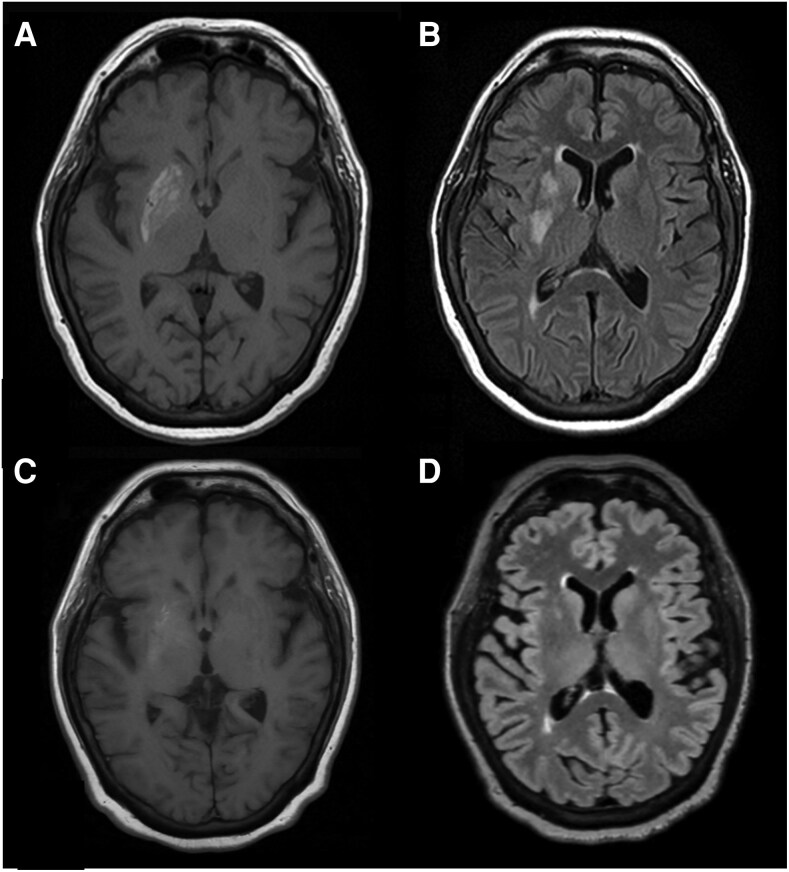
Magnetic resonance imaging of the brain demonstrating T1 hyperintensity (A) and increased signal on T2 FLAIR (B) of the right lentiform nucleus, with near-complete resolution of T1 signal (C) and full resolution of T2 FLAIR signal (D) on three-month follow-up imaging.

A 68-year-old woman with type 2 diabetes presented to the hospital with a 2-month history of intermittent left upper and lower body “writhing” movements, which had resolved spontaneously 1-2 weeks prior to admission. During the symptomatic period, the patient-reported capillary glucose levels were around 400 mg/dL (22.2 mmol/L) but improved to 100-200 mg/dL (5.6-11.1 mmol/L) following significant dietary modification. Hemoglobin A1c (A1c) at admission was 10.7%. Blood glucose levels throughout hospitalization maintained between 90 and 120 mg/dL (5.0-6.7 mmol/L) without the need of insulin, consistent with recently improved glycemic control in the ambulatory setting after dietary modification. Magnetic resonance imaging (MRI) demonstrated T1 hyperintensity of the right lentiform nucleus (Fig. 1A), corresponding to increased signal on T2 fluid-attenuated inversion recovery (FLAIR) sequences (Fig. 1B).

Follow-up MRI performed three months later demonstrated substantially improved mild intrinsic T1 hyperintensity in the right lentiform nucleus (Fig. 1C) and interval resolution of T2 FLAIR hyperintensity (Fig. 1D), which corresponded to improved glycemic control, with A1c 6.1% at that time.

The patient's presentation is consistent with diabetic striatopathy, a rare condition associated with nonketotic hyperglycemia and reversible striatal T1 hyperintensity. Management centers on optimization of glycemic control, though anti-chorea medications may be required in select cases [1].
